# *Demodex folliculorum* 

**DOI:** 10.3390/diagnostics15121520

**Published:** 2025-06-15

**Authors:** Ayyad Zartasht Khan, Fredrik Fineide, Jens Wohlmann, Kjell Gunnar Gundersen, Morten Gundersen, Miriam Kolko, Tor Paaske Utheim

**Affiliations:** 1 Department of Ophthalmology, Østfold Hospital Trust, 1524 Moss, Norway; 2 Department of Ophthalmology, Sørlandet Hospital Trust, 4838 Arendal, Norway; 3 Department of Medical Biochemistry, Oslo University Hospital, 0372 Oslo, Norway; 4 The Norwegian Dry Eye Clinic, 0368 Oslo, Norway; 5 Department of Plastic and Reconstructive Surgery, Oslo University Hospital, 0372 Oslo, Norway; 6 Electron Microscopy Laboratory, Department of Biosciences, University of Oslo, 0316 Oslo, Norway; 7 Ifocus Eye Clinic, 5531 Haugesund, Norway; 8 Department of Ophthalmology, Copenhagen University Hospital, Rigshospitalet-Glostrup, 2600 Glostrup, Denmark; 9 Department of Ophthalmology, Drammen Hospital, Vestre Viken Trust, 3004 Drammen, Norway; 10 Department of Ophthalmology, Oslo University Hospital, 0372 Oslo, Norway; 11 Department of Ophthalmology, Stavanger University Hospital, 4011 Stavanger, Norway; 12 Department of Ophthalmology, Vestfold Hospital Trust, 3103 Tønsberg, Norway; 13 Department of Clinical Medicine, Faculty of Medicine, University of Bergen, 5021 Bergen, Norway; 14 Department of Research and Development, Oslo Metropolitan University, 0130 Oslo, Norway; 15 National Centre for Optics, Vision and Eye Care, Department of Optometry, Radiography and Lighting Design, Faculty of Health Sciences, University of South-Eastern Norway, 3616 Kongsberg, Norway

**Keywords:** *Demodex folliculorum*, scanning electron microscope, dry eye disease, diagnostics

## Abstract

Herein, we present scanning electron microscopy imagery of *Demodex folliculorum* on the eyelashes of a patient with a two-year history of dry, burning, and watery eyes. *Demodex* mites are part of the normal human skin flora, inhabiting hair follicles and sebaceous glands. However, in some individuals, they may contribute to ocular surface diseases, including blepharitis and dry eye disease. Symptoms often include itching, photophobia, and a foreign body sensation. The pathogenic role of *Demodex* is not fully understood but may involve microabrasions, gland obstruction, hypersensitivity reactions, and bacterial dysbiosis. The presence of collarettes at the base of eyelashes is a diagnostic hallmark. Although optimal treatment remains debated, options include topical tea tree oil, ivermectin, and a recently FDA-approved drug lotilaner. Our patient responded favorably to a two-month regimen of tea tree oil-based eyelid wipes. This case underscores the clinical relevance of *Demodex* infestation in chronic ocular discomfort and highlights the importance of diagnostics.

**Figure 1:**
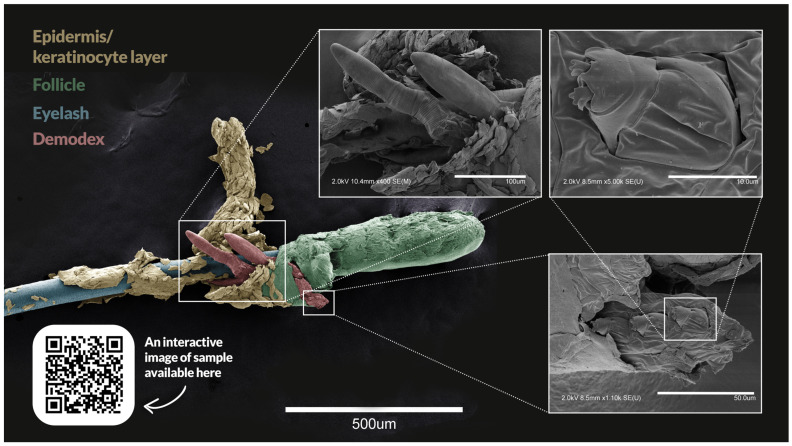
*Demodex* is a genus of parasitic mites; *D. folliculorum* and *D. brevis* are the two species found in humans, inhabiting hair follicles and sebaceous glands, respectively [1]. The adult mites measure approximately 0.3–0.4 mm and complete their life cycle in about 2–3 weeks [2]. Although they are considered part of the normal human microbiota, their overpopulation or aberrant immune response to their presence can lead to clinical disease [3]. In humans, the mites can contribute to the inflammatory process, and hence worsen diseases such as dry eye disease, blepharitis, acne, rosacea, seborrheic dermatitis, and perioral dermatitis [4]. In dogs, *D. canis* can cause a skin condition that leads to patchy hair loss [5]. The scanning electron micrograph above shows *Demodex folliculorum* on the eyelash of a patient who presented with dry, burning, and watery eyes, persisting over the past two years. The image has been digitally colored for clarity: the hair follicle is green, the eyelash is blue, skin cells are yellow, and the three hair follicle mites are highlighted in red. The inset in the upper right corner shows one of the organism’s eight extremities. Diagnostic methods for *Demodex* depend on disease location and include dermoscopy, slit lamp examination, and microscopy techniques for evaluating eyelashes, skin scrapings, or adhesive tape taken from the skin surface [4]. For ocular demodicosis, the presence of collarettes is a pathognomonic sign [6]. Collarettes are waxy, cylindrical crusts around the bases of eyelashes. They consist of undigested material, keratinized cells, mites, and eggs. The use of polymerase chain reaction (PCR) as a diagnostic tool is also gaining interest, but is not as widespread in use in clinical practice as the more conventional methods described earlier [7]. In the case presented herein, the diagnosis of ocular demodicosis was made clinically based on the presence of typical symptoms (ocular surface discomfort, foreign body sensation, and excessive tearing) along with slit-lamp examination findings of collarettes. Although *Demodex* are considered part of the normal flora, they may contribute to or exacerbate common ocular conditions such as blepharitis, dry eyes, tear film instability, ocular rosacea, meibomian gland dysfunction, and chalazion. Symptoms depend on the diagnosis but commonly include itchy eyes (particularly along the lash line), photophobia, watery eyes, and foreign body sensation. The mechanism by which the mites contribute to disease remains debated. Hypotheses include direct damage through microabrasions, blockage of meibomian glands, induction of hypersensitivity and inflammation, and bacterial dysbiosis, as the mites act as reservoirs for bacteria (particularly *Streptococcus* and *Staphylococcus*) [3]. Despite its high prevalence, especially in older populations, *Demodex* remains underdiagnosed. A high index of suspicion should be maintained in patients with treatment-resistant blepharitis or unexplained ocular surface symptoms. The optimal treatment for *Demodex* remains a subject of ongoing debate. Options that have demonstrated efficacy in studies include ivermectin (oral or topical) [8], metronidazole (oral or topical) [9], tea tree oil (topical) [10], Intense Pulsed Light (IPL) therapy [11], and microblepharoexfoliation [3]. Topical lotilaner (a parasite-specific GABA-ergic chloride channel inhibitor) was recently approved by the U.S. Food and Drug Administration (FDA) for the treatment of *Demodex blepharitis* [12]. Our patient experienced relief from symptoms following treatment with tea tree oil-based eyelid wipes used twice daily for two months. This case underscores the clinical relevance of *Demodex* mites in chronic ocular surface disease. In patients presenting with persistent, non-resolving blepharitis or dry eye symptoms, clinicians should consider *Demodex* infestation in their differential diagnosis. Recognition of collarettes and appropriate diagnostic confirmation can facilitate timely and targeted therapy, improving patient outcomes and quality of life. An interactive image of the sample is available here: https://wohlmann.github.io/202403_Demodex/ (accessed on 10 June 2025). Methods: The sample was fixed for 48 h in 4% formaldehyde and 0.8% glutaraldehyde in 1 × PHEM buffer (60 mM PIPES, 25 mM HEPES, 10 mM EGTA, 2 mM MgCl_2_, pH 6.9) [13]. It was then quenched in 100 mM glycine prepared in 100 mM HEPES (4-(2-hydroxyethyl)-1-piperazineethanesulfonic acid) buffer at pH 7.2, dehydrated in an ascending ethanol series (20 min in each concentration of 50, 70, 80, 90, and 96%, followed by two 60 min steps in 100% ethanol), and subsequently subjected to critical point drying (CPD) using a BAL-TEC CPD 030 apparatus (BAL-TEC AG, Balzers, Liechtenstein) through eight cycles and overnight pressure release. The dried sample was mounted on carbon adhesive tape and sputter-coated with a 7 nm layer of platinum using a Cressington Coating System 308R (Cressington Scientific Instruments, Watford, UK). Specimens were examined and imaged using a Zeiss Gemini 300 Field Emission Scanning Electron Microscope (FE-SEM; Zeiss, Oberkochen, Germany). Image stitching, projection and visualization was performed as described previously [14].
